# Comparison of monoclonal gammopathy of undetermined significance-associated neuropathy and chronic inflammatory demyelinating polyneuropathy patients

**DOI:** 10.1007/s00415-014-7357-0

**Published:** 2014-05-07

**Authors:** Nuha M. Alkhawajah, Samantha K. Dunnigan, Vera Bril

**Affiliations:** 1 Department of Medicine (Neurology), King Saud University, Riyadh, Saudi Arabia; 2 Department of Medicine (Neurology), University of Toronto, Toronto, Canada; 3 Toronto General Hospital, Eaton Centre Wing, 5-309, 200 Elizabeth Street., Toronto, ON M5G 2C4 Canada

**Keywords:** Monoclonal gammopathy of undetermined significance, Chronic inflammatory demyelinating polyneuropathy, Demyelinating neuropathy, Axonal neuropathy, Treatment

## Abstract

**Objectives:**

There are varying reports on whether monoclonal gammopathy of undetermined significance-associated neuropathy (MGUSN) patients are distinguishable from those with chronic inflammatory demyelinating polyneuropathy (CIDP) and whether specific MGUSN subclasses are associated with specific clinical phenotypes.

**Methods:**

We performed a retrospective chart review of MGUSN (*n* = 56) and CIDP (*n* = 67) patients. Data extracted included: demographics, neurological examination, and nerve conduction studies (NCS) at baseline and last visit. Clinical status was rated as 0 = worse, 1 = unchanged, 2 = stabilized after a declining course, or 3 = improved. The electrophysiology data were rated as 0 = worse, 1 = stable, or 2 = improved. Statistical analyses were performed using JMP (version 9.0.2 for Macintosh, from SAS).

**Results:**

Seventy percent were males, aged 68.1 ± 12.6 years with neuropathy for 9.8 ± 6.8 years and follow-up of 4.0 ± 3.2 years. CIDP patients had more severe neuropathy, and were more likely to receive treatment and to respond. The clinical neuropathy status remained unchanged in 52.8 % of the MGUSN and 24.2 % of the CIDP patients, and stabilized in 7.6 % of MGUSN and 30.3 % of CIDP patients. IgM-MGUSN patients did not differ from other immunoglobulin subclasses in response to treatment. The clinical severity and the number of abnormal NCS parameters were greater in the demyelinating MGUSN in comparison to the axonal group.

**Conclusion:**

MGUSN patients have less severe neuropathy than CIDP patients, but among the MGUSN patients the severity is greater in the demyelinating and the IgM groups. MGUSN patients may do well without treatment and exposure to potential adverse effects.

## Introduction

Monoclonal gammopathies are a group of disorders characterized by excessive amounts of serum immunoglobulins produced by proliferation of a single clone of plasma cells [1]. Most of the patients have monoclonal gammopathy of undetermined significance (MGUS) and the rest have systemic diseases such as amyloidosis, multiple myeloma, osteosclerotic myeloma, lymphoma, Waldenstrom’s macroglobulinemia, POEMS syndrome (polyneuropathy, organomegaly, endocrinopathy, monoclonal gammopathy and skin changes) and cryoglobulinemia [2–4]. Ten percent of patients with idiopathic neuropathy have monoclonal gammopathy, in contrast to 2.5 % of patients with non-idiopathic neuropathy suggesting an adverse effect of the gammopathy on peripheral nerve function [5]. The most frequent type of immunoglobulin seen with MGUS is IgG, and this is in contrast to MGUS-associated neuropathy (MGUSN) in which the most common type is IgM [3, 6]. MGUSN, classified into IgM and non-IgM associated [7], is an under diagnosed entity [8]. Chronic inflammatory demyelinating polyneuropathy (CIDP) is an acquired symmetrical progressive relapsing sensorimotor neuropathy, and it is reported that 22–30 % of CIDP patients have MGUS [9–11]. There are varying reports on whether MGUSN patients are distinguishable from those with CIDP based on their clinical presentation, electrophysiologic findings and response to treatment [9–12].

We aimed to determine whether the clinical characteristics and response to treatment differed in MGUSN, or any subclass of MGUSN, and CIDP patients.

## Research design and methods

### Subjects

MGUSN and CIDP subjects attending the Neuromuscular Clinic at the University Health Network for management of their immune-mediated polyneuropathy between 1990 and 2013 were evaluated for this study. Our current study involved the extraction of demographic data, clinical history, physical examination, laboratory test results and electrophysiologic data from previously coded charts of MGUSN and CIDP patients. The Research Ethics Board at the University Health Network approved the current study protocol.

All subjects were ≥18 years of age and had a diagnosis of CIDP or MGUSN. The 67 patients with CIDP comprised a cohort matched for age and gender to a CIDP plus diabetes patient cohort from a prior study [13]. The CIDP cohort was selected from a total of 1,900 patients with this diagnosis and was fully representative of the larger cohort. The MGUSN cohort included all patients diagnosed with this disorder in the study period. CIDP was diagnosed based on the clinical presentation, as judged by a neuromuscular expert [VB], and the presence of demyelination on nerve conduction studies (NCS). CIDP patients fulfilled the Koski criteria (chronic polyneuropathy progressive for at least 8 weeks, no serum paraprotein and no genetic abnormality and either recordable compound muscle action potentials in at least 75 % of motor nerves and either abnormal distal latency or abnormal motor conduction velocity or abnormal *F* wave latency in >50 % of motor nerves, OR, symmetric onset or symmetric examination findings and weakness in all four limbs and proximal weakness in at least one limb) [14]. Although these criteria were described later, the CIDP patients in this study fulfilled these criteria and were matched for age and gender to an existing cohort of diabetes patients with CIDP. MGUSN was diagnosed on identification of serum monoclonal proteins by immunoelectrophoresis after exclusion of plasma cell dyscrasias (multiple myeloma, osteosclerotic myeloma, POEM’s syndrome, lymphoma, Waldenstrom’s macroglobulinemia, amyloidosis) by a hematologist, and other possible etiologies of peripheral neuropathy with evaluation of FBS, HbA1c, 2-h GTT, CBC, ESR, anti-GM1 Ganglioside antibodies, LFTs, creatinine, vitamin B12, C3, C4, rheumatoid factor, anti-DS DNA, VDRL and in some cases Lyme serology, West Nile virus, CSF protein and cell count analysis.

### Evaluation

All subjects were evaluated by neurological examination, the validated Toronto Clinical Neuropathy Score (TCNS) [15, 16], vibration perception threshold (VPT), and median; peroneal; tibial and sural NCS.

NCS were performed using the Sierra Wave Electromyography Instrument (Cadwell Laboratories Inc., Kennewick, WA, USA). Age- and height-adjusted NCS reference values were used, according to the standards of the Toronto General Hospital (TGH) University Health Network (UHN) electrophysiologic laboratory.

### Nerve conduction studies

Median, peroneal, tibial and sural NCS were performed using surface stimulating and recording techniques according to the standards of the Canadian Society of Clinical Neurophysiologists and the American Association of Neuromuscular and Electrodiagnostic Medicine [17, 18]. The electromyography instrument measured distal latencies (DL) and amplitudes, and calculated conduction velocities (CV) automatically. Compound muscle action potential (CMAP) amplitude was measured as baseline to peak for the median, peroneal and tibial nerves. For the sural sensory nerve action potential (SNAP), the amplitude was measured as baseline to negative peak, or from the positive peak (if present) to the negative peak. The sural nerve latency was measured at onset from the initial deflection from the baseline. The *F* wave latency was determined as the minimum reproducible latency obtained after 10 supra-maximal stimuli were applied.

At each subsequent visit, patients were assessed by history, clinical examination and repeat NCS. Change in polyneuropathy status was judged on both clinical and electrophysiologic grounds. Using the clinical data from the history and neurological examination at the last visit, the patients were rated as 0 = worse, 1 = unchanged, 2 = stabilized after declining course, or 3 = improved. Using the electrophysiology data, the patients were rated as follows: 0 = worse, 1 = stable or 2 = improved.

### Statistical analyses

Statistical analyses were performed using JMP (version 9.0.2 for Macintosh, from SAS). Demographic data were expressed as mean ± standard deviation (SD) for normally distributed data, or median and interquartile range (IQR) for non-parametric data. Differences in categorical variables were assessed using the *χ* ^2^, while differences in continuous variables were assessed using the ANOVA. *p* values <0.05 were considered significant.

## Results

A total of 123 subjects with a mean age of 68.1 ± 12.6 years were entered into the study. The demographic profile of the patients is shown in Table 1. About 70 % of the patients were males in both groups. The mean duration of neuropathy was 9.8 ± 6.8 years and of follow-up was 4.0 ± 3.2 years. Neuropathy was more severe in those with CIDP as demonstrated by the findings of more abnormality of upper limb reflexes (score of 3 vs 0, *p* = 0.005), lower limb reflexes (score of 6 vs 4, *p* = 0.009) and also more generalized weakness on clinical examination with 28.8 % of CIDP patients having generalized weakness compared with 5.6 % of those with MGUSN (*p* = 0.002) although the severity of weakness was similar (Table 1). However, the percentage of those able to ambulate without aids did not differ between groups with about 75 % of patients freely ambulatory. The greater severity of neuropathy in CIDP patients was evidenced by more abnormal nerve conduction parameters (median nerve CMAP amplitude and CV, peroneal nerve CV and tibial nerve *F* wave latency) as shown in Table 2. This table shows only those NCS parameters that were significantly different, but all other NCS parameters tended to be worse in the CIDP group although not reaching a *p* value of <0.05 (data not shown). Interestingly, lower limb VPT was more abnormal in the MGUSN group.

**Table 1:** Demographic profile of 123 patients with monoclonal gammopathy of undetermined significance-associated neuropathy (MGUSN) (56) and chronic inflammatory demyelinating polyneuropathy (CIDP) (67)

Parameter	MGUSN	CIDP	*p* value^a^
Age (years), mean ± SD	70 ± 11.5	66.5 ± 13.4	0.124
Age range (years)	(45–90)	(36–90)	–
Gender male (%)	70	72	0.808
Duration of follow-up (years), mean ± SD	4.5 ± 0.39	3.3 ± 0.44	0.037
Duration of peripheral neuropathy (years), mean ± SD	9.9 ± 6.1	9.7 ± 7.4	0.920
TCNS_symptoms^b^, Median (IQR)	3 (2–4)	4 (3–5)	0.181
TCNS_sensory^c^, Median (IQR)	4 (2–5)	4 (2–4)	0.496
TCNS_deep tendon reflexes^d^, Median (IQR)	4 (1.25–6)	6 (4–8)	0.009
TCNS_total^e^, Median (IQR)	11 (7–14)	13 (8–16)	0.053
Weakness on exam^f^, *n* (%)	22 (39.3)	34 (50.8)	0.203
Weakness proximal^f^, *n* (%)	3 (5.4)	3 (4.5)	1
Weakness distal^f^, *n* (%)	14 (25)	16 (23.9)	0.886
Weakness generalized^f^, *n* (%)	3 (5.6)	19 (28.8)	0.002
Gait abnormal, *n* (%)	30 (53.6)	34 (50.8)	0.755
Independent walking (%)	78.6	74.6	0.61
Treated patients, *n* (%)	29 (51.8)	62 (92.5)	<0.0001
IVIG, *n* (%)	15 (26.8)	58 (86.6)	<0.0001
Prednisone, *n* (%)	12 (21.4)	44 (65.7)	<0.0001
Plasmapheresis, *n* (%)	10 (17.9)	10 (14.4)	0.661
Azathioprine, *n* (%)	5 (8.9)	36 (53.7)	<0.0001
Mycophenolate mofetil, *n* (%)	2 (3.6)	9 (13.4)	0.065
Rituximab, *n* (%)	3 (5.4)	2 (3)	0.659
Cyclophosphamide, *n* (%)	1 (1.8)	2 (3.0)	1
Methotrexate, *n* (%)	0 (0)	1 (1.5)	1
Chlorambucil, *n* (%)	1 (1.8)	0 (0)	0.459

*IVIG* intravenous immunoglobulin

^a^ *p* values <0.05 are considered significant

^b^Toronto clinical neuropathy score_symptoms: present = 1; absent = 0 (0–6)

^c^Toronto clinical neuropathy score_sensory: abnormal = 1; normal = 0 (0–5)

^d^Toronto clinical neuropathy score_deep tendon reflexes: absent = 2; reduced = 1; normal = 0 (0–8)

^e^Toronto clinical neuropathy score_total: normal = 0 to maximum of 19

^f^Weakness determined by Medical Research Council (MRC) grading of muscles

**Table 2:** Quantitative sensory threshold and nerve conduction testing in 123 patients with monoclonal gammopathy of undetermined significance-associated neuropathy (MGUSN) (56) and chronic inflammatory demyelinating polyneuropathy (CIDP) (67)

Parameter	MGUSN^a^	CIDP^a^	*p* value^b^
VPT-right upper limb	6.20 ± 3.54 (1.7–23.3)	6.32 ± 5.28 (2–39)	0.894
VPT-left upper limb	6.13 ± 3.56 (1.8–21.8)	6.23 ± 5.33 (2–39.7)	0.912
VPT-right lower limb	31.33 ± 15.75 (4–50)	24.07 ± 13.87 (3.2–50)	0.011
VPT-left lower limb	30.41 ± 15.6 (4.2–50)	23.9 ± 13.6 (3.2–50)	0.020
Median CMAP Wrist, mV	9.7 ± 5.3 (0–30.5)	7.45 ± 4.4 (0–19.9)	0.012
Median CMAP Elbow, mV	8.8 ± 5.3 (0–28.7)	6.54 ± 4.2 (0–19.4)	0.011
Median CV, m/s	46.9 ± 9.9 (12–63)	42.1 ± 10.6 (8–60.4)	0.013
Peroneal CV Fibular head, m/s	37 ± 8.8 (7–51)	33.7 ± 7.4 (15–47)	0.049
Peroneal CV Popliteal fossa, m/s	39.4 ± 9 (11–55)	35.41 ± 7.70 (15–53)	0.021
Tibial *F* wave latency, ms	60.9 ± 7.6 (45.6–77.2)	66.6 ± 6.7 (53.8–86.3)	0.008

*VPT* vibration perception threshold, *CMAP* compound muscle action potential amplitude, *CV* conduction velocity

^a^Values are shown as means ± standard deviations (range)

^b^ *p* values <0.05 are considered significant

Ninety-two percent of the CIDP patients received treatment in comparison to 52 % of the MGUSN patients (*p* < 0.0001). Eighty-six percent of the CIDP patients compared with 26.8 % of MGUSN received intravenous immunoglobulin (IVIG), 65.7 % CIDP patients versus 21.4 % MGUSN prednisone, 53.7 % CIDP patients versus 8.9 % MGUSN azathioprine, 14.4 % CIDP patients versus 17.9 % MGUSN plasmapheresis, 13.4 % CIDP patients versus 3.6 % MGUSN mycophenolate mofetil, 3.0 % CIDP patients versus 5.4 % MGUSN rituximab, 3 % CIDP patients versus 1.8 % MGUSN cyclophosphamide, 1.5 % CIDP patients versus 0 MGUSN methotrexate, and 0 CIDP versus 1.8 % MGUSN chlorambucil. CIDP patients were more likely to receive IVIG, prednisone, and azathioprine (*p* < 0.0001), and were more likely to respond to IVIG (*p* < 0.0001). There was no difference in the response to other treatments between the MGUSN and CIDP patients. The clinical neuropathy status stabilized in 7.6 % of MGUSN and 30.3 % of CIDP (*p* = 0.001), and remained unchanged in more than half of the MGUSN and in 24.2 % of the CIDP patients (*p* = 0.001). There was no difference in the percentage of patients who improved or worsened in both groups. The NCS remained stable in the majority of MGUSN and CIDP patients.

In the MGUSN group, 46.4 % had IgM, 39.3 % had IgG, 9 % had IgA, 3.6 % had a combination, and in 1.8 % the immunoglobulin type was unspecified. Forty-five percent of the MGUSN had kappa light chains. The patients were classified according to the heavy chain into two groups: IgM-MGUSN (*n* = 26) and non-IgM-MGUSN with IgG, IgA, or a combination (*n* = 30). The two groups were the same in age, gender, duration of neuropathy and the distribution of light chains (kappa or lambda). Anti-MAG antibodies were seen in 30.8 % of IgM-MGUSN patients and in none of the non-IgM-MGUSN patients (*p* = 0.001). IgM-MGUSN patients had more severe neuropathy as demonstrated by higher VPT values in both upper and lower limbs and a tendency to more abnormal NCS parameters, significantly so for the median and peroneal nerve distal motor latencies. There was no difference between the two groups in distribution of weakness. About ½ the patients were treated in each group. There was no difference in the use of azathioprine, mycophenolate mofetil, rituximab, cyclophosphamide, methotrexate, IVIG or plasmapheresis between the two groups. Prednisone was used more commonly in non-IgM-MGUSN patients (*p* = 0.025). There was no difference in the response to any of the treatments. The clinical neuropathy status remained unchanged in about half of both groups. The NCS remained stable in about two-thirds of the patients in both groups. However, there was a trend towards more clinical and electrophysiologic improvement and less worsening in IgM-MGUSN patients. When comparing IgM-MGUSN patients to CIDP patients, there was no difference in severity (comparable TCNS and NCS with the exception of longer tibial nerve *F* wave latencies in the CIDP group), but VPT was higher in those with IgM paraproteins. Comparing non-IgM-MGUSN to CIDP patients, CIDP patients had more severe neuropathy as demonstrated by both TCNS and NCS.

We stratified the MGUSN patients into demyelinating (*n* = 29) and axonal (*n* = 27) forms as shown in Table 3. We found that those classified as demyelinating had more lambda light chains (14 vs 5, *p* = 0.017), had all the anti-MAG antibody-positive patients (8 vs 0, *p* = 0.005) and had more severe polyneuropathy as shown by the VPT, TCNS data and the number of abnormal NCS parameters (Table 3). There was no difference in age, gender or duration of neuropathy.

**Table 3:** Patients with monoclonal gammopathy of undetermined significance-associated neuropathy (MGUSN) classified as demyelinating or axonal polyneuropathy

Parameter	Demyelinating	Axonal	*p* value^a^
Gender male (%)	69	70.4	0.91
Age (years), mean ± SD	70 ± 11.4	70 ± 11.7	0.982
Duration of follow-up in years, mean ± SD	3.2 ± 2.7	3.3 ± 3.5	0.913
IgG, *n* (%)	9 (31)	13 (48.2)	0.189
IgM, *n* (%)	17 (58.6)	9 (33.3)	0.057
IgA, *n* (%)	2 (6.9)	3 (11.1)	0.58
IgM and IgG, *n* (%)	0	1 (3.7)	0.482
IgG and IgA, *n* (%)	0	1 (3.7)	0.482
Unspecified, *n* (%)	1 (3.5)	0	1
Lambda, *n* (%)	14 (48.3)	5 (18.5)	0.017
Kappa, *n* (%)	10 (34.5)	15 (55.6)	0.112
Lambda and kappa, *n* (%)	1 (3.5)	5 (18.5)	0.096
Unspecified, *n* (%)	4 (13.8)	2 (7.4)	0.671
Anti-MAG, *n* (%)	8 (27.6)	0	0.005
VPT-right upper limb, mean ± SD	7.2 ± 4.5	5.1 ± 1.7	0.343
VPT-left upper limb, mean ± SD	7.2 ± 4.5	5.0 ± 1.7	0.029
VPT-right lower limb, mean ± SD	38.4 ± 14.6	24.0 ± 13.7	0.001
VPT-left lower limb, mean ± SD	37.4 ± 14.5	23.9 ± 14.0	0.001
TCNS-symptoms^b^, mean ± SD	3.7 ± 1.4	3.0 ± 1.3	0.059
TCNS-sensory^c^, mean ± SD	3.8 ± 1.3	2.8 ± 1.9	0.026
TCNS-deep tendon reflexes^d^, mean ± SD	4.7 ± 2.8	3.1 ± 2.6	0.037
TCNS-total^e^, mean ± SD	12.1 ± 3.8	8.9 ± 4.1	0.004
Number of abnormal nerve conduction studies parameters	8.1 ± 2.5	5.7 ± 3.5	0.005

*MAG* myelin associated glycoprotein, *VPT* vibration perception threshold

^a^ *p* values <0.05 are considered significant

^b^Toronto clinical neuropathy score_symptoms: present = 1; absent = 0 (0–6)

^c^Toronto clinical neuropathy score_sensory: abnormal = 1; normal = 0 (0–5)

^d^Toronto clinical neuropathy score_deep tendon reflexes: absent = 2; reduced = 1; normal = 0 (0–8)

^e^Toronto clinical neuropathy score_total: normal = 0 to maximum of 19

## Discussion

We examined our cohort of MGUSN patients and compared them to an existing cohort of CIDP patients to determine whether the clinical characteristics and response to treatment differed between the groups or in subclasses of MGUSN.

The age, gender and the duration of the polyneuropathy did not differ between the two groups in this study, similar to findings in a previous report [10]. However, other authors found that CIDP-MGUS patients were more likely to be older males in comparison to idiopathic CIDP (CIDP-I) [11, 12] .

In our cohort, CIDP patients had more severe neuropathy based on both clinical and electrodiagnostic measures. These differences were most apparent between non-IGM-MGUSN and CIDP patients (data not shown) than between IgM-MGUSN and CIDP patients. Several studies compared CIDP patients with and without MGUS [9–12], but none—up to our knowledge—compared purely axonal MGUSN to CIDP or subclasses of MGUSN to CIDP. Our results are similar to those of Simmons et al. [9] who found less severe weakness and a more indolent course in CIDP-MGUS. However, they contrast with Gorson et al. [10] who found that CIDP-MGUS patients did not have a more indolent course despite the fact that weakness was less common in this group, and Bromberg et al. who found that CIDP-MGUS cannot be distinguished from CIDP-I on the basis of NCS [11]. In our cohort, we found differences but particular cut-off values for individual patients would require a different study. About 90 % of the CIDP patients in comparison to 50 % of the MGUSN patients in our cohort were treated, likely due to greater severity of the disease in the CIDP patients warranting treatment. More CIDP patients were stabilized compared to MGUSN patients, but there were no differences in patients categorized as improving or worsening. Because of the uneven number of treated patients in both groups, we examined the outcome of the treated MGUSN patients only and compared it to the treated CIDP patients. We found the post treatment clinical and electrophysiologic status to be the same in both groups, except that more treated CIDP patients stabilized in comparison to treated MGUSN patients (32.8 versus 10.3 %, respectively, *p* = 0.037). The CIDP patients had more responders to IVIG treatment when compared to all MGUSN patients combined or when compared to those with IgM- or non-IGM-MGUSN patients, and had more responders to plasmapheresis when compared to non-IgM-MGUSN patients in contrast to an earlier report [10]. In a long-term follow-up by Simmons et al. [12] in which 90 % of the CIDP-I and 80 % of the CIDP-MGUS patients were treated, CIDP-MGUS had more functional impairment at the end of the follow-up period, but we do not find a similar outcome in this study.

It is thought that MGUSN patients have demyelinating disease and that axonal features are secondary [19], but Notermans et al. [20] found that 40 % of MGUSN are purely axonal. Filostso et al. [8] detected pure axonal findings in 46 % of IgG and 20 % of IgM-MGUSN patients. In our cohort, about 48 % of the MGUSN patients had primarily axonal neuropathy and 52 % were primarily demyelinating. The lambda paraprotein was observed more frequently in the demyelinating category and all anti-MAG antibody-positive patients fell into this group, although the prevalence of anti-MAG was less than described by Filosto et al. [8]. Although there were no differences in the gender, age of onset or duration of the peripheral neuropathy, the clinical severity and the number of abnormal NCS parameters were greater in the demyelinating group as was the percentage of treated patients (70 % of the demyelinating vs 30 % of the axonal, *p* = 0.001). In our cohort, axonal and demyelinating MGUSN patients responded similarly to treatment in contrast to the experience of Gorson et al. [19].

As reported previously [21–23], there were no differences between subclasses of MGUSN (IgM versus others) with respect to duration of peripheral neuropathy, age, gender or presenting symptoms, or signs. However, others found that IgM-MGUSN patients are more likely to have sensory dysfunction, progressive course, and a more severe predominantly demyelinating polyneuropathy in comparison to other subclasses [20–22, 24, 25]. On NCS we found that IgM-MGUSN group had longer distal motor latencies in the upper and lower limbs similar to the findings of Suarez and Kelly [24]. There was no difference in response to treatment between the two groups as reported previously by Simovic et al. [21], this is in contrast to Yeung et al. [23] who concluded that IgG- and IgA-MGUSN patients are more likely to respond to treatment than IgM-MGUSN patients.

Our results support the concept that CIDP patients differ from those with MGUSN. The MGUSN clinical phenotype shows milder disease that leads less frequently to treatment although IgM-MGUSN patients had more severe disease within the MGUSN group. Further, demyelinating MGUSN patients had more severe clinical disease with significantly more abnormal NCS parameters in comparison to axonal MGUSN. As well, treatment response rate appears to be lower in MGUSN patients, adding some validity to the choice to defer treatment in these patients. Given the potential adverse effects of most immunosuppressive treatments, and the expense and limited access to immunomodulation treatments, it is important to determine the best therapeutic choices for patients with immune-mediated polyneuropathies. Our study suggests that those with MGUSN may do well without treatment in many cases and patients can avoid exposure to adverse effects and high costs. Even with treatment, it should be noted that only few patients stabilized or improved.

The limitations of our study are that it is a retrospective review with inherent difficulties in standardized evaluations, treatments, and data collection; however, all reports comparing MGUSN to CIDP—up to our knowledge—have been retrospective to date [9–12, 26]. Also, the CIDP cohort was selected for a prior study for age and gender factors, and not all patients diagnosed with CIDP in our clinic within the timeframe of the study were included and thus the results may not be generalizable to all CIDP patients. Some novel and improved measures in inflammatory neuropathies such as the Rasch overall disability scale [27] and the overall neuropathy limitation scale (ONLS) [28] were not available when these patients were evaluated and their use might have provided better insight into patient outcomes. In addition, the TCNS is not a linear scale and sensitivity to change may be limited. Finally, MGUSN patients had milder disease with a floor effect for improvement. Further, although we defined the demyelinating categories on the basis of NCS, we do not have morphological confirmation of the underlying pathology. Despite these considerations, our study suggests that MGUSN differs from CIDP both in clinical severity and response to treatment. MGUSN is a heterogenous disorder and alone it shows variability between IgM and non-IgM forms. A multi-center prospective natural history and intervention study might allow better determination of which MGUSN patients to treat.
